# Spectral Entropy Monitoring Accelerates the Emergence from Sevoflurane Anesthesia in Thoracic Surgery: A Randomized Controlled Trial

**DOI:** 10.3390/jcm11061631

**Published:** 2022-03-15

**Authors:** Jui-Tai Chen, Yu-Ming Wu, Tung-Yu Tiong, Juan P. Cata, Kuang-Tai Kuo, Chun-Cheng Li, Hsin-Yi Liu, Yih-Giun Cherng, Hsiang-Ling Wu, Ying-Hsuan Tai

**Affiliations:** 1 Department of Anesthesiology, Shuang Ho Hospital, Taipei Medical University, New Taipei City 23561, Taiwan; 19240@s.tmu.edu.tw (J.-T.C.); 15538@s.tmu.edu.tw (Y.-M.W.); 15193@s.tmu.edu.tw (C.-C.L.); 18384@s.tmu.edu.tw (H.-Y.L.); stainless@s.tmu.edu.tw (Y.-G.C.); 2 Department of Anesthesiology, School of Medicine, College of Medicine, Taipei Medical University, Taipei 11031, Taiwan; 3 Division of Thoracic Surgery, Department of Surgery, Shuang Ho Hospital, Taipei Medical University, New Taipei City 23561, Taiwan; 09262@s.tmu.edu.tw (T.-Y.T.); ktkuo@tmu.edu.tw (K.-T.K.); 4 Division of Thoracic Surgery, Department of Surgery, School of Medicine, College of Medicine, Taipei Medical University, Taipei 11031, Taiwan; 5 Department of Anesthesiology and Perioperative Medicine, The University of Texas MD Anderson Cancer Center, 1515 Holcombe Blvd, Unit 409, Houston, TX 77030, USA; jcata@mdanderson.org; 6 Department of Anesthesiology, Taipei Veterans General Hospital, Taipei 11217, Taiwan; hlwu9@vghtpe.gov.tw; 7 School of Medicine, National Yang Ming Chiao Tung University, Taipei 11221, Taiwan

**Keywords:** chest surgery, delirium, depth of anesthesia, electroencephalography, emergence agitation

## Abstract

The clinical efficacy of spectral entropy monitoring in improving postoperative recovery remains unclear. This trial aimed to investigate the impact of M-Entropy (GE Healthcare, Helsinki, Finland) guidance on emergence from anesthesia and postoperative delirium in thoracic surgery. Adult patients undergoing video-assisted thoracoscopic surgery for lung resection at a medical center were randomly allocated into the M-Entropy guidance group (*n* = 39) and the control group (*n* = 37). In the M-Entropy guidance group, sevoflurane anesthesia was titrated to maintain response and state entropy values between 40 and 60 intraoperatively. In the control group, the dosing of sevoflurane was adjusted based on clinical judgment and vital signs. The primary outcome was time to spontaneous eye opening. M-Entropy guidance significantly reduced the time proportion of deep anesthesia (entropy value <40) during surgery, mean difference: −21.5% (95% confidence interval (CI): −32.7 to −10.3) for response entropy and −24.2% (−36.3 to −12.2) for state entropy. M-Entropy guidance significantly shortened time to spontaneous eye opening compared to clinical signs, mean difference: −154 s (95% CI: −259 to −49). In addition, patients of the M-Entropy group had a lower rate of emergence agitation (absolute risk reduction: 0.166, 95% CI: 0.005–0.328) and delirium (0.245, 0.093–0.396) at the postanesthesia care unit. M-Entropy-guided anesthesia hastened awakening and potentially prevented emergence agitation and delirium after thoracic surgery. These results may provide an implication for facilitating postoperative recovery and reducing the complications associated with delayed emergence and delirium.

## 1. Introduction

In thoracic surgery, the procedure of thoracotomy results in pneumothorax and many physiology disturbances, including ventilation–perfusion abnormalities and hypoxia [1,2]. Additionally, one-lung ventilation (OLV) is a common method to facilitate surgical exposure in video-assisted thoracic surgery. Nevertheless, OLV potentially produces significant respiratory and hemodynamic changes, including pulmonary barotrauma and profound hypotension [1,2]. Hemodynamically unstable patients are predisposed to a nonoptimal depth of anesthesia and relevant complications, such as delayed emergence and postoperative delirium [3,4]. Despite recent advances in surgical and anesthesia techniques, patients are particularly susceptible to delayed and complicated recovery following thoracic surgery due to invasive procedures and concomitant morbidities [5,6].

Electroencephalography-based monitoring enables real-time measurements of anesthetic depth and guides the use of intravenous or inhalational general anesthetics during surgery [7,8]. Mounting evidence has indicated that bispectral index guidance might accelerate the return of consciousness from general anesthesia among surgical patients compared to usual practice [9]. However, it remains unclear whether spectral entropy monitoring facilitates emergence from general anesthesia and protects against postoperative delirium following thoracic surgery [10,11,12,13,14,15,16]. Previous studies focused on abdominal [11,15], gynecological [11,12], orthopedic [11,13], cardiac [14], and miscellaneous types of surgery [10,16]. Little is known about the clinical benefits of spectral entropy guidance on anesthetic dosing in the recovery after thoracic surgery.

In this randomized controlled trial, we aimed to investigate the putative impact of spectral entropy monitoring on the emergence from sevoflurane anesthesia and the rate of delirium in patients following thoracic surgery. In this study, an M-Entropy™ module (GE Healthcare, Helsinki, Finland) was used to continuously evaluate depth of anesthesia. Based on the current evidence, we hypothesized that M-Entropy guidance shortened times to emergence from anesthesia and reduced postoperative delirium in thoracic surgery [8,9,10,11,12,13,14,15,16].

## 2. Materials and Methods

### 2.1. Clinical Settings

This trial was reviewed and approved by the research ethics committee of Taipei Medical University in Taiwan (TMU-JIRB-N202004045). It was registered in an international online registry, ClinicalTrials.gov (Identifier: NCT04414228; date of registration: 4 June 2020). All participants gave written informed consents before randomizations. All research methods were conducted in accordance with the standards of the Declaration of Helsinki and related regulations. This trial conformed to the recommendations of the Consolidated Standards of Reporting Trials (CONSORT) statement [17].

**Figure 1:**
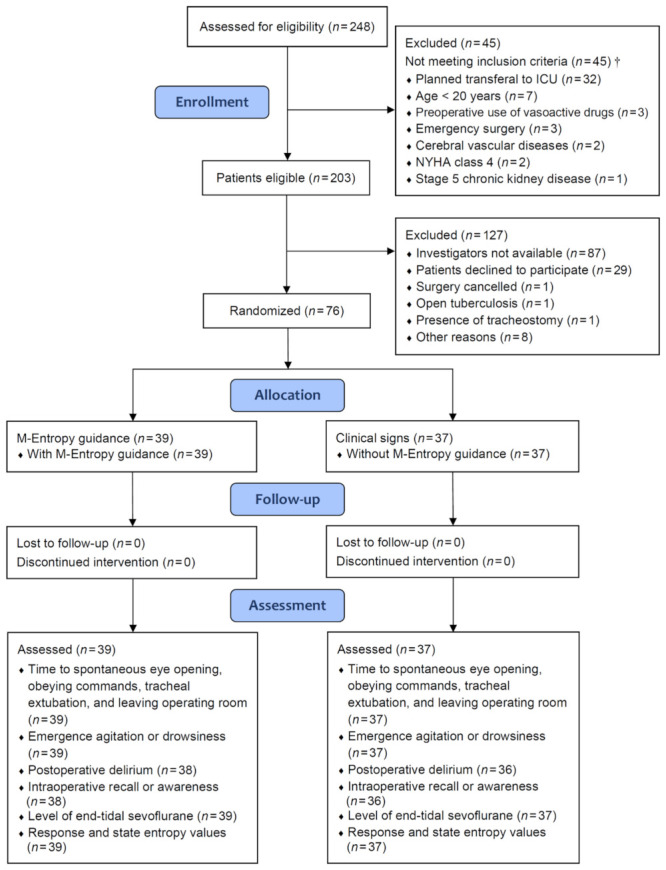
Consolidated Standards of Reporting Trials flow diagram. † Not mutually exclusive because patients can have more than one exclusion criterion.

We enrolled patients who underwent video-assisted thoracoscopic surgery for lung resections at Shuang Ho Hospital, Taipei Medical University, from June 2020 to October 2021. Exclusion criteria included age <20 years, cerebral vascular disease or trauma, use of sedative or antipsychotic drugs within preoperative 30 days, stage 5 chronic kidney disease (estimated glomerular filtration rate <15 mL·min·1.73 m^−2^), New York Heart Association functional class 4, presence of circulatory shock needing vasoactive agents before surgery, emergency surgery, planned postoperative admission to intensive care unit for mechanical ventilation, pregnancy, and patient refusal (Figure 1). All surgeries were performed by the same team of physicians, using the same method of surgery.

### 2.2. Randomization Process

In this prospective, two-arm, randomized controlled trial, patients were randomized into two groups in a 1:1 ratio: the M-Entropy guidance group or the clinical sign group. We used the random-number function of statistical software to generate random permuted blocks of four. After giving the informed consent, each subject was assigned a unique identifier and a group allocation by the principal investigator (Y.-H.T.). The assignment was enclosed in an opaque envelope. After patients arrived at the operating theater, an independent attending anesthesiologist (J.-T.C. or C.-C.L.) opened the prepared envelope and delivered the assigned intervention.

### 2.3. Anesthesia Protocol

Patients were given fentanyl 1–2 μg·kg^−1^ and propofol 1–2 mg·kg^−1^ for induction of anesthesia. An intravenous bolus of rocuronium 0.8 mg·kg^−1^ was used to facilitate the placement of a 35 or 37 Fr double-lumen endotracheal tube (Mallinckrodt Endobronchial Tube, Covidien, Ireland). Sevoflurane was used to maintain general anesthesia with a fresh gas flow of 6 L·min^−1^ during the first 5 min and 1.5–2 L·min^−1^ thereafter. The vaporizer was set at 2 vol% during the first 5 min. An intravenous bolus of fentanyl 50 μg was administered before the surgical incision. Pressure-controlled ventilation was utilized with a peak pressure lower than 30 cm H_2_O and a positive end-expiratory pressure of 5 cm H_2_O. To maintain peripheral oxygen saturation ≥ 92%, the inspiratory oxygen fraction of 60% was applied during two-lung ventilation, and 80–100% was used during OLV. For two-lung ventilation, tidal volumes up to 8 mL·kg^−1^ and a respiratory rate of 10–15 min^−1^ were used to ensure end-tidal carbon dioxide below 45 mm Hg during surgery. For OLV, tidal volumes up to 6–7 mL·kg^−1^ with a respiratory frequency of 10–15 min^−1^ were used. In all patients, sevoflurane was discontinued after the wound closure, and the fresh gas flow was increased to 6 L·min^−1^ with an inspiratory oxygen fraction of 100%. Once the train-of-four count recovered to ≥1, patients were decurarized using sugammadex dosed at 2 mg·kg^−1^. To maintain end-tidal carbon dioxide lower than 45 mm Hg, manual ventilation was applied until patients regained spontaneous ventilation. On-demand intravenous analgesia with morphine 3–10 mg and/or ketorolac 15–30 mg was used to relieve postoperative pain at the postanesthesia care unit (PACU) [18].

### 2.4. Spectral Entropy Monitoring and Guidance

Before induction of anesthesia, an M-Entropy sensor (GE Healthcare, Helsinki, Finland) was applied to the patient’s forehead according to the manufacturer’s recommendations. The M-Entropy monitor was concealed from the patients and surgeons. In the M-Entropy guidance group, the dosing of sevoflurane was titrated to maintain the response and state entropy numbers between 40 and 60 throughout the surgery. In the clinical sign group, sevoflurane anesthesia was adjusted based on vital signs and clinical judgment. This was typically to maintain heart rate and mean arterial pressure within the 20% range of the baseline values. In case of inadequate anesthesia signs (e.g., swallowing, movement, and cough), the dosage of sevoflurane was increased. M-Entropy monitoring was continued in the clinical sign group, but the entropy numbers were concealed from the anesthetists. The data of entropy numbers and expiratory gas were recorded in a 5 min interval.

### 2.5. Study Outcome

The primary outcome was time to spontaneous eye opening, defined as the time from cessation of sevoflurane to patients’ spontaneous eye opening. The secondary outcomes were time to obeying verbal commands (sustained handgrip or head lift for 5 s), time to tracheal extubation, and time to leaving the operating theater. We also evaluated emergence agitation and drowsiness during extubation, postoperative delirium, and intraoperative recall or awareness. We used the Richmond Agitation-Sedation Scale (RASS) to quantify the level of consciousness [19]. Agitation was defined as a RASS score +2 to +4 and drowsiness as −2 to −5. The Confusion Assessment Method was used to evaluate delirium 30 min after the arrival at the PACU and 2 h after transferal to the ward [20]. Intraoperative recall or awareness was assessed with a modified Brice structured interview during the first postoperative day [21]. For patients with an unanticipated transferal to the intensive care unit, postoperative delirium and intraoperative recall or awareness were not assessed. An independent attending anesthesiologist (Y.-M.W.), blinded to group allocation, determined times to emergence from anesthesia, evaluated the level of consciousness during extubation, and adjudicated postoperative delirium and intraoperative recall or awareness. At the PACU, medical and nursing staff were masked to group allocation.

### 2.6. Statistical Analysis

A prior meta-analysis demonstrated that the mean difference in time to awakening was 5.42 min (approximately 325 s) between the M-Entropy group and the standard practice group [22]. Accordingly, at least 12 patients in each group of M-Entropy guidance and clinical signs are needed to detect a difference of 325 s in time to spontaneous eye opening between groups, accepting a type I error of 5% and type II error of 10% with anticipated time to spontaneous eye opening of mean 550 ± standard deviation (SD) 250 s in the clinical sign group [22,23]. After data were tested for normality of distribution using the Shapiro–Wilk W test and Kolmogorov–Smirnov test, normally distributed data were expressed as mean ± SD. Non-normally distributed variables were expressed as median with interquartile range and range. Baseline patient attributes and study outcomes were compared between the two groups using either independent t tests or Wilcoxon rank-sum tests for continuous data and χ^2^ tests or Fisher exact tests for categorical data, as appropriate. Intention-to-treat analysis was used for all study outcomes. A two-sided level of 0.05 was considered statistically significant. All statistical analyses were conducted using Statistics Analysis System (SAS), version 9.4 (SAS Institute Inc., Cary, NC, USA).

## 3. Results

### 3.1. Baseline Characteristics of the Participants

A total of 76 patients were randomized into the M-Entropy guidance group (*n* = 39) and the clinical sign group (*n* = 37). The distributions of demographics, coexisting diseases, pulmonary and cardiac functions, and laboratory test results were generally balanced between the two groups (Table 1). The distributions of surgical types, baseline entropy values, doses of intravenous anesthetics, and intraoperative fluid volumes were also comparable between the two groups, except for a slightly higher dose of fentanyl in the M-Entropy guidance group (Table 2). There was no difference in surgical blood loss or anesthesia duration between groups. One patient in the M-Entropy guidance group and one in the clinical sign group had an unplanned admission to the intensive care unit for mechanical ventilation. Intraoperative vital signs were generally comparable between groups (Supplementary Table S1).

### 3.2. Depth of Anesthesia

Patients in the M-Entropy guidance group had a higher time percentage of response and state entropy values ranged from 40 to 60 compared to those in the clinical sign group, mean difference: 19.1% (95% confidence interval (CI): 8.3–30.0, *p* = 0.0008) for response entropy and 21.8% (95% CI: 10.6–33.0, *p* = 0.0003) for state entropy. The time percentage of response and state entropy values below 40 was significantly reduced by M-Entropy guidance, mean difference: –21.5% (95% CI: −32.7 to −10.3, *p* = 0.0004) for response entropy and –24.2% (95% CI: −36.3 to −12.2, *p* = 0.0002) for state entropy). The average response and state entropy values were significantly higher in the M-Entropy guidance group compared to the clinical sign group (Table 3).

### 3.3. Recovery from Anesthesia and Postoperative Delirium

Patients receiving M-Entropy guidance had a significantly shorter time to spontaneous eye opening compared to those of the clinical sign group, mean difference: −154 s (95% CI: −259 to −49, *p* = 0.0047) (Table 4). Times to obeying verbal commands, tracheal extubation, and leaving the operating room were comparable between the two groups. M-Entropy guidance was also associated with a lower incidence of emergence agitation, absolute risk reduction: 0.166 (95% CI: 0.005 to 0.328, *p* = 0.0469) and number needed to treat: 7. In addition, the risk of postoperative delirium was significantly lower in the M-Entropy guidance group compared to the clinical sign group, absolute risk reduction: 0.245 (95% CI: 0.093 to 0.396, *p* = 0.0024) and number needed to treat: 5. There was no intraoperative awareness or recall reported by the participants.

## 4. Discussion

In this randomized clinical trial, we found that spectral entropy guidance on sevoflurane dosing significantly shortened time to awakening and reduced the rates of emergence agitation and delirium after pulmonary resection. The beneficial effects were related to decreased time proportions of deep anesthesia during surgery. To our knowledge, the present study was the first to specifically evaluate the potential benefits of M-Entropy-guided anesthesia in facilitating emergence from anesthesia in thoracic surgery. These findings may provide a clinical implication for mitigating the adverse effects of delayed emergence and delirium and improving postoperative recovery following thoracic surgery.

There are few studies investigating the effect of spectral entropy monitoring on emergence from anesthesia among adult surgical patients [10,11,12,13,14,15,16]. Preceding studies included patients undergoing abdominal [11,15], gynecological [11,12], orthopedic [11,13], cardiac [14], and mixed types of surgery [10,16]. Furthermore, most studies used propofol-based intravenous general anesthesia rather than inhalational anesthesia [10,12,13,14,16]. Therefore, it is difficult to generalize these results to patients receiving inhalational general anesthesia for thoracic surgery. Our results indicated that M-Entropy guidance was significantly associated with faster awakening, in line with three studies [10,12,16] but not another [13]. Differences in surgical types, anesthetic protocols, and patient characteristics were possibly responsible for the discrepancies. Dinu and colleagues recently reported that use of spectral entropy monitoring to optimize the dosage of anesthetics reduced the incidences of hypotension and bradycardia during surgery [3]. However, our study did not observe a significant difference in intraoperative hemodynamic parameters between the two groups. The discrepancy might result from the differences in surgical types and outcome definitions. Of note, most of our patients were at low risk in terms of age, cardiopulmonary comorbidities, and baseline functional capacity. This might conceal the potential benefits of spectral entropy monitoring in maintaining hemodynamic stability and accelerating postoperative recovery. Importantly, our trial demonstrated that M-Entropy guidance might prevent the occurrence of emergence agitation and delirium, which was not shown in previous studies [10,11,12,13,14,15,16].

The Enhanced Recovery After Surgery (ERAS) Society and the European Society of Thoracic Surgeons recently recommended a standard anesthetic protocol to improve recovery after lung surgery, including lung-protective ventilation during one-lung ventilation, coadministrations of regional and general anesthesia, and short-acting volatile or intravenous anesthetics [24]. However, the use of electroencephalography-guided anesthesia was neglected in the guideline [24]. The present study indicated that an optimal range of anesthetic depth using the M-Entropy system could accelerate the return of consciousness and reduce the risk of emergence agitation and postoperative delirium in the setting of lung surgery. Although the difference in awakening times between the two groups was only modest, these results might add important evidence to the ERAS protocol by elucidating the efficacy of continuous spectral entropy monitoring for optimizing anesthetic depth during thoracic surgery.

The incidence of delirium after thoracic surgery was reported to range from 5.3% to 6.7%, which was lower compared to that of our study [25,26]. This might be due to the retrospective nature of prior studies [25,26]. Postoperative delirium represents an important risk factor for both cognitive deficits and physical dysfunction after surgery [27,28]. It has been reported that deep anesthesia and electroencephalogram suppression were associated with the development of postoperative delirium [29]. However, it remains controversial whether anesthetic depth monitoring protects against postoperative delirium and cognitive function decline, with benefits reported in some studies [30,31,32] but not in others [33,34]. These contrasting results might be explained by the differences in the patient attributes, anesthetic regimens, and protocol rigor [30,31,32,33,34]. Given the multiple contributing causes and inadequate understanding of pathophysiology, it is challenging to prevent or treat postoperative delirium in the high-risk population [35]. More efforts are required to explore the interactions between delirium and other comorbidities.

There were some limitations to this study. First, the number of subjects in this trial was small, which might generate underpowered statistics. Second, the consumption of sevoflurane and the cost of general anesthesia were not evaluated in this trial [3,11,15]. Therefore, we could not examine the cost and benefit of M-Entropy guidance. Third, this trial only assessed postoperative delirium during the first postoperative day. Consequently, it remains unclear whether M-Entropy monitoring prevents delirium or cognitive decline throughout the postoperative hospital stay. Fourth, M-Entropy guidance reduced time to awakening by merely 2–3 min in this study. The clinical significance of spectral entropy monitoring in daily practice awaits further investigations. Fifth, we did not evaluate the intraoperative nociceptive state (e.g., surgical pleth index and analgesia nociception index) and postoperative pain scores. Therefore, it remains unknown whether M-Entropy guidance influences postoperative pain intensity and thereby modifies the development of delirium. Finally, this is a single-center study, and the results may not be applicable to hospitals with different clinical settings.

## 5. Conclusions

Intraoperative M-Entropy guidance on optimizing anesthetic depth effectively accelerated the emergence from sevoflurane anesthesia and decreased the rates of emergence agitation and postoperative delirium among patients undergoing pulmonary resection. These findings were related to a light depth of anesthesia and reduced anesthetic exposure. Our results might provide a clinical implication for improving the recovery after thoracic surgery. Future studies should focus on patients at high risk of delayed emergence and delirium, such as those who have preexisting cognitive deficits or prolonged general anesthesia. Furthermore, large clinical trials are warranted to better understand the potential effect of M-Entropy guidance on postanesthesia recovery.

## Figures and Tables

**Table 1:** Baseline characteristics of enrolled patients.

	Entropy Guidance *n* = 39		Clinical Signs *n* = 37		*p*
Age, year	59.8	15.7	60.2	15.6	0.9122
Sex, male	18	46.2	19	51.4	0.6505
Body mass index, kg·m^−2^	23.9	22.4–26.2 (16.6–35.6)	23.0	21.3–25.2 (17.6–39.2)	0.2797
ASA physical status					>0.9999
I	3	7.7	3	7.7	
II	36	92.3	34	91.9	
Current cigarette smoking	11	28.2	10	27.0	0.9086
Current alcohol drinking	4	10.3	2	5.4	0.6752
Lung malignancy	14	35.9	12	32.4	0.7503
Coexisting disease					
Hypertension	9	23.1	16	43.2	0.0614
Diabetes mellitus	5	12.8	4	10.8	>0.9999
Ischemic heart disease	3	7.7	5	13.5	0.4747
COPD	0	0	4	10.8	0.0515
Chronic kidney disease	1	2.6	1	2.7	>0.9999
Liver disease	4	10.3	1	2.7	0.3589
Carotid arterial disease	0	0	1	2.7	0.4868
Pulmonary function test					
FVC, % predicted	88.0	78.2–103.9 (56.3–127.3)	81.3	76.8–93.0 (54.2–158.4)	0.3501
FEV1, % predicted	87.5	73.5–95.9 (49.0–131.1)	84.5	71.6–93.7 (40.3–162.3)	0.6979
Left ventricular ejection fraction, %	67	7	70	8	0.1341
Preoperative blood test					
Hemoglobin, g·dL^−1^	13.9	13.0–14.5 (9.0–15.8)	13.1	11.7–14.4 (10.6–17.2)	0.0553
Creatinine, mg·dL^−1^	0.80	0.64–0.94 (0.36–1.35)	0.86	0.79–1.08 (0.51–1.80)	0.0762
eGFR, mL·min·1.73 m^−2^	90.1	77.0–108.8 (53.2–198.9)	80.0	65.8–103.0 (37.0–130.7)	0.1515
Urea nitrogen, mg·dL^−1^	14	12–18 (6–27)	15	13–19 (9–23)	0.4323
Sodium, mmol·L^−1^	139	138–141 (135–143)	139	138–140 (129–145)	0.3705
Potassium, mmol·L^−1^	3.8	3.6–4.0 (3.1–4.4)	3.8	3.6–4.1 (2.8–4.9)	0.7575
Alanine aminotransferase, U·L^−1^	22	17–28 (12–53)	22	17–27 (11–59)	0.9202
Aspartate aminotransferase, U·L^−1^	22	20–24 (11–80)	21	18–27 (11–95)	0.9349

Values are mean ± standard deviation, median with interquartile range (range), or counts with percent. Abbreviations: ASA, American Society of Anesthesiologists; COPD, chronic obstruction pulmonary disease; eGFR, estimated glomerular filtration rate; FEV1, forced expiratory volume in one second; FVC, forced vital capacity.

**Table 2:** Surgical and anesthetic parameters.

	Entropy Guidance *n* = 39		Clinical Signs *n* = 37		*p*
Surgical procedures					0.9642
Wedge resection	22	56.4	22	59.5	
Segmentectomy	9	23.1	8	21.6	
Lobectomy	8	20.5	7	18.9	
RE value before induction	98	95–99 (71–100)	97	96–98 (90–100)	0.8004
SE value before induction	87	86–89 (70–91)	88	86–89 (79–91)	0.5127
Intravenous anesthetics					
Fentanyl, μg	150	125–175 (50–250)	125	100–150 (50–200)	0.0320
Propofol, mg	120	110–150 (70–200)	105	100–140 (60–300)	0.1327
Rocuronium, mg	90	60–110 (40–140)	80	70–100 (40–140)	0.7226
Sugammadex, mg	130	120–160 (100–200)	130	120–150 (90–200)	0.7810
Amount of crystalloid fluids, mL	700	500–900 (350–1500)	800	600–1000 (400–1400)	0.1754
Amount of colloid fluids, mL	0	0–0 (0–500)	0	0–0 (0–500)	0.2584
Surgical blood loss, mL	10	10–50 (10–350)	10	10–100 (10–500)	0.1641
Duration of anesthesia, min	190	135–285 (75–400)	210	145–247 (80–505)	0.9917

Values are median with interquartile range (range) or counts with percent. Abbreviations: RE, response entropy; SE: state entropy.

**Table 3:** End-tidal sevoflurane levels and entropy values.

	Entropy Guidance *n* = 39		Clinical Signs *n* = 37		*p*
Average level of end-tidal sevoflurane, %	1.49	1.19–1.72 (0.72–2.08)	1.58	1.45–1.68 (1.10–2.06)	0.2660
Average level of end-tidal sevoflurane, aaMAC	0.78	0.61–0.92 (0.43–1.17)	0.86	0.73–0.95 (0.58–1.19)	0.1499
Time percentage of RE > 60, %	14.5	6.7–30.0 (2.7–73.1)	11.3	5.8–23.1 (1.8–75.0)	0.3496
Time percentage of RE 40–60, %	77.8	65.5–88.2 (26.9–96.1)	71.1	29.1–76.9 (0–90.9)	0.0056
Time percentage of RE < 40, %	1.9	0–6.0 (0–29.2)	10.0	3.6–40.6 (0–97.4)	0.0002
Average RE value	55	51–59 (44–69)	51	44–57 (26–68)	0.0117
Time percentage of SE > 60, %	13.8	4.7–18.5 (0–65.4)	9.4	4.3–14.3 (0–66.7)	0.2142
Time percentage of SE 40–60, %	81.5	72.3–90.0 (34.6–96.1)	71.8	35.4–82.2 (0–90.9)	0.0020
Time percentage of SE < 40, %	2.3	0–7.5 (0–46.2)	13.2	4.2–46.9 (0–97.4)	0.0001
Average SE value	52	49–57 (41–65)	49	42–54 (26–65)	0.0093

Values are median with interquartile range (range). Abbreviations: aaMAC, age-adjusted minimum alveolar concentration; RE, response entropy, SE: state entropy.

**Table 4:** Emergence from anesthesia and postoperative delirium.

	Entropy Guidance *n* = 39		Clinical Signs *n* = 37		*p*
Time to spontaneous eye opening, s	427	270–530 (56–830)	505	395–736 (209–1226)	0.0155
Time to obeying commands, s	506	388–644 (95–1201)	550	445–823 (215–1310)	0.1006
Time to tracheal extubation, s	565	420–840 (135–7116)	595	504–863 (256–11,927)	0.1685
Time to leaving operating room, s	885	804–1103 (450–1744)	1030	882–1200 (575–1590)	0.1178
Emergence agitation	3	7.7	9	24.3	0.0469
Drowsiness during tracheal extubation	2	5.1	6	16.2	0.1481
Postoperative delirium	1	2.6	10	27.0	0.0024
Intraoperative awareness or recall	0	0	0	0	NA

Values are counts with percent or median with interquartile range (range). Abbreviations: NA, not applicable.

## Data Availability

The datasets of this study are available from the corresponding author on reasonable request. The data are not publicly available due to the regulations of the research ethics committee.

## Supplementary Materials

The following supporting information can be downloaded at: https://www.mdpi.com/article/10.3390/jcm11061631/s1, Table S1: Intraoperative hemodynamic parameters.

## References

[1] Lumb A.B., Slinger P. (2015). Hypoxic pulmonary vasoconstriction: Physiology and anesthetic implications. Anesthesiology.

[2] Campos J.H., Feider A. (2018). Hypoxia during one-lung ventilation: A review and update. J. Cardiothorac. Vasc. Anesth..

[3] Dinu A.R., Rogobete A.F., Popovici S.E., Bedreag O.H., Papurica M., Dumbuleu C.M., Velovan R.R., Toma D., Georgescu C.M., Trache L.I. (2020). Impact of general anesthesia guided by state entropy (SE) and response entropy (RE) on perioperative stability in elective laparoscopic cholecystectomy patients: A prospective observational randomized monocentric study. Entropy.

[4] Thomas E., Martin F., Pollard B. (2020). Delayed recovery of consciousness after general anaesthesia. BJA Educ..

[5] Hung M.H., Chen J.S., Cheng Y.J. (2019). Precise anesthesia in thoracoscopic operations. Curr. Opin. Anaesthesiol..

[6] Pedoto A., Perrino A.C. (2019). Delayed recovery following thoracic surgery: Persistent issues and potential interventions. Curr. Opin. Anaesthesiol..

[7] Liang Z., Wang Y., Sun X., Li D., Voss L.J., Sleigh J.W., Hagihira S., Li X. (2015). EEG entropy measures in anesthesia. Front. Comput. Neurosci..

[8] Wu Y.M., Su Y.H., Huang S.Y., Lo P.H., Chen J.T., Chang H.C., Yang Y.L., Cherng Y.G., Wu H.L., Tai Y.H. (2021). Recovery profiles of sevoflurane and desflurane with or without M-Entropy guidance in obese patients: A randomized controlled trial. J. Clin. Med..

[9] Lewis S.R., Pritchard M.W., Fawcett L.J., Punjasawadwong Y. (2019). Bispectral index for improving intraoperative awareness and early postoperative recovery in adults. Cochrane Database Syst. Rev..

[10] Vakkuri A., Yli-Hankala A., Sandin R., Mustola S., Høymork S., Nyblom S., Talja P., Sampson T., van Gils M., Viertiö-Oja H. (2005). Spectral entropy monitoring is associated with reduced propofol use and faster emergence in propofol-nitrous oxide-alfentanil anesthesia. Anesthesiology.

[11] Aimé I., Verroust N., Masson-Lefoll C., Taylor G., Laloë P.A., Liu N., Fischler M. (2006). Does monitoring bispectral index or spectral entropy reduce sevoflurane use?. Anesth. Analg..

[12] Gruenewald M., Zhou J., Schloemerkemper N., Meybohm P., Weiler N., Tonner P.H., Scholz J., Bein B. (2007). M-Entropy guidance vs standard practice during propofol-remifentanil anaesthesia: A randomised controlled trial. Anaesthesia.

[13] Ellerkmann R.K., Soehle M., Riese G., Zinserling J., Wirz S., Hoeft A., Bruhn J. (2010). The Entropy Module and Bispectral Index as guidance for propofol-remifentanil anaesthesia in combination with regional anaesthesia compared with a standard clinical practice group. Anaesth. Intensive Care.

[14] Jiahai M., Xueyan W., Yonggang X., Jianhong Y., Qunhui H., Zhi L., Juan D., Xiuliang J. (2012). Spectral entropy monitoring reduces anesthetic dosage for patients undergoing off-pump coronary artery bypass graft surgery. J. Cardiothorac. Vasc. Anesth..

[15] El Hor T., Van Der Linden P., De Hert S., Mélot C., Bidgoli J. (2013). Impact of entropy monitoring on volatile anesthetic uptake. Anesthesiology.

[16] Gruenewald M., Harju J., Preckel B., Molnár Z., Yli-Hankala A., Rosskopf F., Koers L., Orban A., Bein B., AoA Study Group (2021). Comparison of adequacy of anaesthesia monitoring with standard clinical practice monitoring during routine general anaesthesia: An international, multicentre, single-blinded randomised controlled trial. Eur. J. Anaesthesiol..

[17] Boutron I., Altman D.G., Moher D., Schulz K.F., Ravaud P., CONSORT NPT Group (2017). CONSORT Statement for randomized trials of nonpharmacologic treatments: A 2017 update and a CONSORT extension for nonpharmacologic trial abstracts. Ann. Intern. Med..

[18] Ling Y.H., Tai Y.H., Wu H.L., Fu W.L., Tsou M.Y., Chang K.Y. (2021). Evaluating the association of preoperative parecoxib with acute pain trajectories after video-assisted thoracoscopic surgery: A single-centre cohort study in Taiwan. BMJ Open.

[19] Ely E.W., Truman B., Shintani A., Thomason J.W., Wheeler A.P., Gordon S., Francis J., Speroff T., Gautam S., Margolin R. (2003). Monitoring sedation status over time in ICU patients: Reliability and validity of the Richmond Agitation-Sedation Scale (RASS). JAMA.

[20] Inouye S.K., van Dyck C.H., Alessi C.A., Balkin S., Siegal A.P., Horwitz R.I. (1990). Clarifying confusion: The confusion assessment method. A new method for detection of delirium. Ann. Intern. Med..

[21] Brice D.D., Hetherington R.R., Utting J.E. (1970). A simple study of awareness and dreaming during anaesthesia. Br. J. Anaesth..

[22] Chhabra A., Subramaniam R., Srivastava A., Prabhakar H., Kalaivani M., Paranjape S. (2016). Spectral entropy monitoring for adults and children undergoing general anaesthesia. Cochrane Database Syst. Rev..

[23] Armitage P., Berry G., Matthews J.N.S., Armitage P., Berry G., Matthews J.N.S. (2002). Sample-Size Determination. Statistical Methods in Medical Research.

[24] Batchelor T.J.P., Rasburn N.J., Abdelnour-Berchtold E., Brunelli A., Cerfolio R.J., Gonzalez M., Ljungqvist O., Petersen R.H., Popescu W.M., Slinger P.D. (2019). Guidelines for enhanced recovery after lung surgery: Recommendations of the Enhanced Recovery After Surgery (ERAS®) Society and the European Society of Thoracic Surgeons (ESTS). Eur. J. Cardiothorac. Surg..

[25] Yildizeli B., Ozyurtkan M.O., Batirel H.F., Kuşcu K., Bekiroğlu N., Yüksel M. (2005). Factors associated with postoperative delirium after thoracic surgery. Ann. Thorac. Surg..

[26] Hayashi K., Motoishi M., Sawai S., Horimoto K., Hanaoka J. (2019). Postoperative delirium after lung resection for primary lung cancer: Risk factors, risk scoring system, and prognosis. PLoS ONE.

[27] Shi Z., Mei X., Li C., Chen Y., Zheng H., Wu Y., Zheng H., Liu L., Marcantonio E.R., Xie Z. (2019). Postoperative delirium is associated with long-term decline in activities of daily living. Anesthesiology.

[28] Goldberg T.E., Chen C., Wang Y., Jung E., Swanson A., Ing C., Garcia P.S., Whittington R.A., Moitra V. (2020). Association of delirium with long-term cognitive decline: A meta-analysis. JAMA Neurol..

[29] Fritz B.A., King C.R., Ben Abdallah A., Lin N., Mickle A.M., Budelier T.P., Oberhaus J., Park D., Maybrier H.R., Wildes T.S. (2020). ENGAGES Research Group. Preoperative cognitive abnormality, intraoperative electroencephalogram suppression, and postoperative delirium: A mediation analysis. Anesthesiology.

[30] Sieber F.E., Zakriya K.J., Gottschalk A., Blute M.R., Lee H.B., Rosenberg P.B., Mears S.C. (2010). Sedation depth during spinal anesthesia and the development of postoperative delirium in elderly patients undergoing hip fracture repair. Mayo Clin. Proc..

[31] Weinstein S.M., Poultsides L., Baaklini L.R., Mörwald E.E., Cozowicz C., Saleh J.N., Arrington M.B., Poeran J., Zubizarreta N., Memtsoudis S.G. (2018). Postoperative delirium in total knee and hip arthroplasty patients: A study of perioperative modifiable risk factors. Br. J. Anaesth..

[32] Evered L.A., Chan M.T.V., Han R., Chu M.H.M., Cheng B.P., Scott D.A., Pryor K.O., Sessler D.I., Veselis R., Frampton C. (2021). Anaesthetic depth and delirium after major surgery: A randomised clinical trial. Br. J. Anaesth..

[33] Wildes T.S., Mickle A.M., Ben Abdallah A., Maybrier H.R., Oberhaus J., Budelier T.P., Kronzer A., McKinnon S.L., Park D., Torres B.A. (2019). ENGAGES Research Group. Effect of electroencephalography-guided anesthetic administration on postoperative delirium among older adults undergoing major surgery: The ENGAGES randomized clinical trial. JAMA.

[34] Tang C.J., Jin Z., Sands L.P., Pleasants D., Tabatabai S., Hong Y., Leung J.M. (2020). ADAPT-2: A randomized clinical trial to reduce intraoperative EEG suppression in older surgical patients undergoing major noncardiac surgery. Anesth. Analg..

[35] Duning T., Ilting-Reuke K., Beckhuis M., Oswald D. (2021). Postoperative delirium: Treatment and prevention. Curr. Opin. Anaesthesiol..

