# Supplementary material for: Spectral Entropy Monitoring Accelerates the Emergence from Sevoflurane Anesthesia in Thoracic Surgery: A Randomized Controlled Trial
Source: J Clin Med. 2022 Mar 15;11(6):1631. doi: 10.3390/jcm11061631 (PMC8948899; doi:10.3390/jcm11061631)
Supplement: Supplementary file 1 [file jcm-11-01631-s001.zip › jcm-1612255-supplementary.pdf]

Supplementary Table S1. Intraoperative hemodynamic parameters

	Entropy guidance n = 39		Clinical signs n = 37		p
**Before induction**					
Heart rate, beat·min^{-1}	77	66 – 89 (41 – 112)	73	68 – 91 (56 – 116)	0.9917
Systolic blood pressure, mm Hg	139	116 – 166 (106 – 199)	152	132 – 171 (107 – 207)	0.1372
Diastolic blood pressure, mm Hg	86	80 – 92 (67 – 117)	84	76 – 95 (60 – 115)	0.3796
Body temperature, °C	36.1	35.9 – 36.5 (35.0 – 38.0)	36.1	35.8 – 36.2 (35.1 – 37.1)	0.2357
SpO_2, %	98	96 – 99 (95 – 100)	97	96 – 98 (94 – 100)	0.0839
**5 min after induction**					
Heart rate, beat·min^{-1}	82	71 – 93 (54 – 122)	88	80 – 98 (59 – 140)	0.0982
Systolic blood pressure, mm Hg	110	98 – 129 (67 – 198)	108	98 – 129 (70 – 233)	0.9172
Diastolic blood pressure, mm Hg	65	58 – 71 (40 – 103)	67	58 – 73 (46 – 138)	0.4924
Body temperature, °C	35.8	35.4 – 36.1 (34.7 – 36.7)	35.8	35.5 – 36.1 (34.5 – 37.1)	0.9792
SpO_2, %	99	99 – 100 (96 – 100)	99	99 – 100 (95 – 100)	0.2369
**5 min after one-lung ventilation**					
Heart rate, beat·min^{-1}	77	67 – 87 (54 – 105)	84	71 – 90 (52 – 118)	0.1667
Systolic blood pressure, mm Hg	115	105 – 137 (90 – 192)	115	102 – 132 (88 – 178)	0.9503
Diastolic blood pressure, mm Hg	67	63 – 74 (48 – 107)	68	60 – 73 (45 – 95)	0.8637
Body temperature, °C	35.7	35.3 – 36.1 (33.9 – 36.7)	35.8	35.5 – 36.0 (34.9 – 37.1)	0.8635
SpO_2, %	100	98 – 100 (94 – 100)	99	99 – 100 (78 – 100)	0.9147
**5 min after chest is open**					
Heart rate, beat·min^{-1}	82	71 – 86 (61 – 101)	83	75 – 91 (61 – 107)	0.4571
Systolic blood pressure, mm Hg	144	126 – 159 (99 – 178)	130	114 – 154 (93 – 204)	0.0984
Diastolic blood pressure, mm Hg	83	72 – 95	77	66 – 89	0.1330

		(57 – 119)		(49 – 110)	
Body temperature, °C	35.6	35.1 – 36.0 (33.8 – 36.6)	35.7	35.3 – 35.9 (34.8 – 37.0)	0.4226
SpO_2, %	98	95 – 100 (72 – 100)	99	97 – 100 (91 – 100)	0.1863
**Cessation of volatile anesthetics**					
Heart rate, beat·min^{-1}	87	72 – 93 (60 – 112)	86	75 – 96 (59 – 115)	0.6141
Systolic blood pressure, mm Hg	137	123 – 154 (100 – 186)	131	119 – 145 (85 – 228)	0.2010
Diastolic blood pressure, mm Hg	74	67 – 83 (56 – 107)	73	64 – 80 (48 – 125)	0.4635
Body temperature, °C	35.7	35.1 – 36.2 (33.9 – 37.0)	35.8	35.4 – 36.4 (34.7 – 37.2)	0.1915
SpO_2, %	100	99 – 100 (72 – 100)	100	99 – 100 (93 – 100)	0.7329
**5 min after tracheal extubation**					
Heart rate, beat·min^{-1}	89	78 – 98 (57 – 123)	85	79 – 100 (59 – 114)	0.8191
Systolic blood pressure, mm Hg	135	125 – 161 (92 – 186)	137	118 – 153 (97 – 179)	0.4604
Diastolic blood pressure, mm Hg	75	70 – 85 (58 – 99)	75	66 – 84 (50 – 104)	0.5191
Body temperature, °C	36.2	35.9 – 36.3 (35.0 – 37.4)	36.3	36.0 – 36.5 (34.7 – 37.7)	0.1939
SpO_2, %	100	98 – 100 (95 – 100)	100	98 – 100 (96 – 100)	0.8660

Values are median with interquartile range (range). Abbreviations: SpO_2, peripheral oxygen saturation by pulse oximetry.
